# Research progress on the effect of sericin on bone regeneration: A review

**DOI:** 10.1097/MD.0000000000050035

**Published:** 2026-08-14

**Authors:** Rui Wang, Jiatong Zou, Jiajun Xin, Yifei Dai, Xiantao Chen, Yushen Li, Bowei Wang, Zhihui Liu

**Affiliations:** a School of Stomatology, Jilin University, Changchun, Jilin, China; b Department of Gynecology and Obstetrics, The Second Hospital of Jilin University, Changchun, Jilin, China; c Hospital of Stomatology, Jilin University, Changchun, Jilin, China.

**Keywords:** bone regeneration, histological engineering, sericin

## Abstract

Sericin is a waste material from the silk production process, researchers its found to have good biological activity and is widely used in tissue engineering. The aim of this paper is to summarize the biological applications of sericin in the field of bone tissue engineering and their research progress. Randomized controlled trials, prospective or retrospective clinical studies, case series and reports, and systematic evaluations. MEDLINE, PubMed, and Google Scholar were searched using keywords. Sericin, has the ability to promote cell proliferation and angiogenesis. It also has excellent bone regenerative properties such as promoting osteoblast differentiation. Sericin itself has excellent physicochemical properties and can be processed into materials with different properties for bone regeneration engineering. Bone defects due to various skeletal diseases or surgical needs, etc are a major challenge for clinical treatment. Modern tissue engineering using synthetic osteogenic active materials offers another therapeutic option for bone defects, and sericin is expected to play a greater role as a promising biological material.

## Graphical abstract

**Figure:**
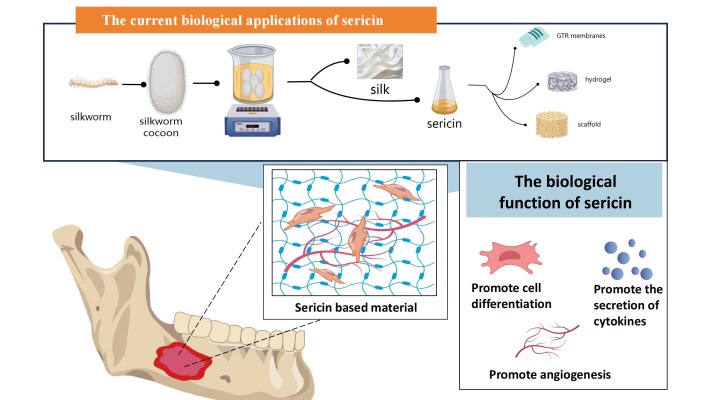

## 1. Introduction

The role of bones in the human body is crucial, attributed to their capability of detoxification and sustenance of soft tissues and their ability to preserve the equilibrium of minerals within the body.^[1]^ Owing to diverse medical conditions, including cancer, infection, trauma, and so on, patients may endure varying degrees of bone deformities, which could disrupt the standard physiological functionality of the bones. The capacity for bones to regenerate is present; however, if the defect surpasses a critical size, this ability to self-heal and restore cannot occur.^[2]^ Surgical implantation of bone grafts, including autologous and allograft bone grafting, is necessary to address significant bone defects. Autologous bone grafting remains the gold standard for treating bone defects.^[3]^ Autologous bone grafts are typically harvested from the ilium, tibia, and fibula, and they are associated with complications and donor site limitations. On the other hand, allogeneic bone grafting involves the potential risks of immune rejection and infection.^[4]^

Bone tissue engineering offers cutting-edge technology for addressing bone defects, effectively integrating material engineering and life sciences to produce bone substitute materials.^[5]^ Bone substitute materials replicate the matrix effect in tissue regeneration, offering mechanical support and fostering a suitable environment for cell adhesion, proliferation, and differentiation.^[6]^ Bone regeneration should exhibit the traits of bone induction, bone conduction, and bone integration. Bone induction refers to the process by which mesenchymal stem cells (MSCs) are derived from host tissues surrounding implants, and induced to proliferate and differentiate into osteoblasts, ultimately leading to the formation of new bone through the action of specific factors. Bone conduction refers to the process where the implant creates a microenvironment that fosters the development of blood vessels and new bones. Bone integration refers to the direct contact between the implant and bone tissue without an intermediate filament layer.^[7]^

Silk, an organic material derived from silkworms, primarily comprises hydrophilic Sericin and hydrophobic silk fibroin. In recent years, researchers have begun exploring its potential applications in the biomedical field.^[8]^ For an extended period, to enhance the commercial value of silk as a textile material, sericin has been commonly discarded as industrial waste during degumming.^[9]^ Silk fibroin demonstrates superior biocompatibility, exceptional mechanical properties, and manageable degradation, making it extensively utilized in various biomedical fields, such as tissue engineering scaffolds, guided bone regeneration membranes, and wound dressings.^[10]^ In recent times, the salutary attributes of sericin have been increasingly investigated and showcased, which include anti-inflammatory, antioxidant, angiogenesis, and osteogenic differentiation activities, among others.^[11]^

## 2. Basic properties of sericin

### 2.1. Regulatory genes of sericin

Sericin is produced from the secretion of glands in Lepidoptera, mainly from the domestic silkworm. The cocoon of the domestic silkworm is formed by the peripheral sericin protein wrapped around the central silk protein. The synthesis of sericin, which is regulated by several genes, has been reported for the 4 sericin genes that constitute the cocoon, *ser1, ser2, ser3,*^[12]^ *and ser4*,^[13,14]^ as well as for a non-cocoon sericin gene, *ser5*.^[15]^ *Ser1* is a full-length, 24-kb, single-copy gene consisting of 9 exons and 8 introns *Ser1* is a full-length 24-kb single-copy gene composed of 9 exons 8 introns that encodes for the production of serine-rich amino acid fragments with a tendency to form β-sheet structures; *ser3* contains approximately 45% of the serine-rich amino acid fragments encoded by ser1 but has a low content of β-sheet structures; *ser2* encodes a protein consisting of a 15-amino acid repeating sequence that is enriched with charged residues such as lysine, aspartic acid, glutamate, and arginine; and *ser4* encodes a protein that is enriched with a large number of charged amino acids and glutamate. The *ser4*-encoded protein is enriched with many charged amino acids and glutamine. *ser5* encodes a non-cocoon sericin protein that is mechanically stronger than cocoon sericin.^[16]^

### 2.2. Structure of sericin

Sericin, a water-soluble globular protein, comprises 18 types of amino acids, with molecular weights ranging from 10 to 400 kDa.^[13]^ Its molecular structure consists of polypeptide chains reiterated in approximately the same sequence, rich in glycine, serine, and other amino acids. Sericin possesses 2 primary structures: α-helix and β-fold. α-Helical sericin molecules form an α-spiral structure, linked by hydrogen bonds within the molecules; β-folded sericin molecules are intersected by a short polypeptide chain and connected to complete molecules through hydrogen bonds and other non-covalent bonds.^[17]^

Different sericin proteins contain other components, which give the fibers they form different physical and mechanical properties. The amino acid content of sericin extracted by various extraction methods was the same.^[18]^ When the raw materials for sericin extraction were cocoon shells and coats, the relative molecular mass distribution range, amino acid composition, and sericin content differed. The relative molecular mass of sericin extracted from the cocoon shell was distributed between 66,200 and 130,000, while the relative molecular mass of sericin extracted from the raw coat was distributed between 43,000 and 130,000. The content of aspartic acid, serine, threonine and glycine in amino acid of the inner coat sericin powder compared with that of the cocoon shell sericin powder was decreased, and the content of glutamic acid and lysine was significantly increased.^[19]^ The amino acid composition of the small molecule protein peptide obtained by neutral protease digestion of cocoon shell sericin was similar to that of the large molecular weight protein peptide, with only a slight decrease in the proportion of serine and an increase in the content of glutamic acid, and the small molecule protein peptide was of the corner structure and “random” curly structure. Sericin with the above molecular structure are metabolized in vivo as peptides and contains amino acids with potential biological functions, such as lysine for its antioxidant effect (inflammation inhibition), aspartic acid and serine, which can play a crucial role in biomineralization.

### 2.3. Biological activity of sericin

#### 2.3.1. Biological safety

Biosafety is one of the essential properties that bioactive materials should have. As a potential biological material, sericin is safe and nontoxic, shows an affinity for tissue cells, and is non-immunogenic in vivo, which are its outstanding advantages. In previous studies, some scientists used a cell counting kit-8 to measure the viability of NIH3T3 fibroblasts and HaCaT keratinocytes incubated with sericin wound dressings, respectively. The results showed no difference between the proliferation of cells in the sericin group and that of the control group,^[20]^ which suggests that sericin is of superior biocompatibility. Maya et al co-cultured sericin with Caco2-BBE cells (epithelioid) and Raw 264.7 macrophages, respectively, and found that the cytotoxicity of sericin was negligible.^[21]^ In one study, sericin was isolated and purified from mutant and wild-type cocoons, respectively, and then injected subcutaneously as a hydrogel into the back of BALB/c mice. The specific IgG levels were similar to those of fibrinogen, an FDA-approved biosafety material.^[22]^ It is essential for the bio-application of sericin, which has been shown not to produce adverse effects on biohazardous substances, both at the cellular tissue level and at the level of individual organs.

As a natural biomaterial, the safety profile of sericin has been validated through numerous in vitro and in vivo studies, with partial clinical applications in humans documented in existing literature. For instance, in clinical trials evaluating sericin-based hydrogels for chronic wound repair, no significant allergic reactions or systemic toxicity were observed in patients, with only 9% experiencing localized adverse reactions (compared to 20% in the control group).^[23]^ Another clinical study reported that wounds treated with sericin-coated materials exhibited a 2% incidence of pruritus during 5-week follow-up assessments, in contrast to 8% observed in the control group.^[24]^ Although existing evidence suggests minimal localized reactions to sericin in humans and limited documentation of associated side effects or adverse events, comprehensive clinical investigations remain imperative to conclusively establish its safety profile prior to broader clinical implementation.

#### 2.3.2. Promote cell proliferation and differentiation

Sericin or materials made of sericin mostly have properties that can promote cell proliferation and differentiation. In ancient China, scraps of silkworm cocoons were used to treat wounds. In contemporary research, some scientists found that undifferentiated mouse stem cells coated with intact sericin substrate proliferation enhancement; some of them then made sericin wound dressings, applied to mouse wound models, and found that this can effectively accelerate the healing of mouse skin wounds and enhance the proliferation of the whole layer of the skin cells.^[21]^

It was found that the molecular weight of sericin also affects its properties. In their study, Cheng Jukang et al extracted freeze-dried sericin through boiling water and distilled water and then dissolved the powder into a sericin solution and co-cultured it with macrophages and adipose stem cells, and found that the low molecular weight sericin treatment could promote the proliferation of macrophages and stimulate the secretion of inflammatory cytokines and chemokines by macrophages. At the same time, the low molecular weight sericin was found to be processed with the adipose stem cells for the first hour. The adipose stem cells proliferated significantly, and there was no significant effect in the subsequent period. Significantly, with no significant impact on the following hours. At a sericin concentration of 1 mg/mL, bone marrow-derived mesenchymal stem cells (BMSCs) demonstrated a proliferation rate of 142 ± 8% over 72 hours (compared to 100% in the control group). However, when the concentration was increased to 5 mg/mL, the proliferation rate decreased to 115 ± 6%, potentially due to inhibitory effects at higher concentrations. Low molecular weight sericin (<30 kDa) significantly enhanced macrophage proliferation by 58 ± 7% within 24 hours, whereas high molecular weight sericin (>100 kDa) only showed a modest increase of 12 ± 3%.^[25]^ The effect of large molecular weight sericin on cell proliferation was relatively weakened, which may be related to the functional groups of sericin playing a pivotal role.

Sericin is a kind of multi-active protein. It has been found that sericin can activate the CCR6-Akt-JNK-NF-κB pathway to enhance cell adhesion. At the same time, sericin can activate cell proliferation through the ccr6-mediated ERK1/2-MAPK pathway,^[26]^ thus promoting cell migration, adhesion, proliferation, and differentiation. To a certain extent, sericin exhibits the role of messenger molecules. However, I believe that how to explain the behavior of sericin in exercising their messenger function will be explored in depth in future studies.

## 3. Development of sericin composites

As an ideal base material for tissue engineering, sericin, in addition to being biocompatible and able to exhibit extracellular matrix-like properties to provide a microenvironment for cell growth, possesses a specific mechanical strength to support cell growth. Biomaterials offer a platform for tissue regeneration in the form of scaffolds, delivery of drugs and provision of growth factors.

### 3.1. Enhancing implant material activity

It has been shown that sericin can cover the surface of other biomaterials to promote cell mitosis^[27]^; for example, it is often added to culture media to enhance cell proliferation and attachment. Sericin M (approximately 400 kDa) extracted from silkworm cocoons significantly enhanced the initial adhesion capacity of human dermal fibroblasts. After 72 hours of culture, viable cell counts reached 250% of the sericin-free control group, with efficacy approaching that of collagen (300%).^[28]^ Sericin is generally used to assist in optimizing the biological activity of other implantable materials by cross-linking with other molecules, for example, on poly lactic acid (PLLA) films by surface modification with sericin using carbodiimide chemistry to enhance the attachment of MSCs to their surfaces. The pure PLLA exhibited a contact angle of 78.38 ± 1.07°, which significantly decreased to approximately 42° (across all concentration groups) post-modification, indicating markedly enhanced hydrophilicity. Quantitative analysis revealed significantly higher MSC attachment in all sericin-modified groups (2.5, 5, and 10 mg/mL), with cell counts ranging from 10.3 × 10^4^ to 10.67 × 10^4^, compared to the pure PLLA group (7.6 × 10^4^, *P* < .05).^[29]^ In addition, coating sericin on poly ε-caprolactone composite scaffolds significantly enhanced cell adhesion while improving MSC differentiation.^[30]^ Sericin can also cover the surface of titanium and its alloy materials to improve titanium implants’ osseointegration and antimicrobial properties, as summarized in Table 1 which includes references to key studies in this area.^[31–36]^

**Table 1:** Surface modification of titanium by sericin.

Modified mode	Modification function	References
Sericin is covalently bound to titanium silicide based on glutaraldehyde	The antibacterial properties of the material were enhanced	^[31]^
Methacrylate-modified titanium surface is chemically coupled with sericin	Reduce bacterial adhesion on its surface; Increase the adhesion, proliferation and activation of alkaline phosphatase of osteoblasts	^[32]^
Some of the tyrosine in sericin oxidized by tyrosinase formed anchor points and were deposited on the surface of the titanium	It prevents bacterial cell adhesion and early biofilm formation	^[33]^
The surface of titanium was fixed with glutaraldehyde as a crosslinking agent	The mRNA expressions of bone salivary protein, osteocalcin and alkaline phosphatase were up-regulated. It has the potential to enhance the osteopathic union	^[34]^
The synthesized alloy was surface modified by matrix-assisted pulsed laser evaporation (MAPLE) technology of graphene nanosheet/sericin (GNP-SS) composite film	The growth and osteogenic differentiation of MC3T3-E1 cells were significantly enhanced. It can regulate the biological activity of osteoblasts and is expected to improve the biological response in vivo	^[35]^
Bioactive nanofiber coatings containing different amounts of *Equisetum arvense* (EA) of polyvinyl alcohol-sericin were obtained by electrospinning	The biological activity of titanium was enhanced. The bone induction rate was improved	^[36]^

### 3.2. Biohydrogels

Hydrogels are a widely used strategy for tissue engineering due to their ideal moisturizing properties and porosity biocompatibility, biodegradability and biomimetic properties. Based on the protein properties of sericin, it can be processed into hydrogel materials through chemical cross-linking,^[37]^ 3D printing^[38]^ and electrostatic spinning,^[39]^ and as a polymeric protein, sericin can be metabolized in vivo and stored and utilized as amino acids. However, this can have limitations; sericin hydrogel materials show low strength and support.^[40]^ Thus, it has been a continuous process to explore how to fully use the properties of sericin and make the materials prepared from it meet the mechanical properties requirements.

Throughout the majority of protein-based materials, scientists are committed to using chemical methods to ensure the original activity of the protein based on the preparation of materials with a specific mechanical strength. Studies have shown that sericin can be rapidly formed by chemical cross-linking agents such as glutaraldehyde^[41]^ and carbodiimide,^[31]^ which can be used as cell carriers in tissue engineering,^[42]^ such as skin tissue engineering,^[43]^ and it is evident that the action of these 2 substances does not affect the original activity of sericin and makes the tensile strength of the material significantly increased. The compressive modulus was measured as 12.5 ± 1.8 MPa, accompanied by a porosity of 85 ± 3% and pore size of 200 ± 50 μm. These parameters satisfy the mechanical requirements for cancellous bone applications (10–50 MPa). In another study, a novel hydrogel with intrinsic neurotrophic and neuroprotective functions was generated by the biocompatible cross-linking agent genipin, which provides an effective cell delivery platform with applicability and potential for brain tissue repair,^[44]^ and this cross-linking method, in turn, makes the sericin material flowable for better neuroprotection. A new study based on methacrylic acid, sericin and gelatin via photo-crosslinking to make hydrogels loaded with human umbilical cord MSCs, which have good biocompatibility, mechanical and degradation properties and have great potential for repairing or regenerating endometrium,^[45]^ such material would balance strength and deformability. A double crosslinked sericin hydrogel with a loose and porous structure enhanced mechanical properties and relatively improved elastic properties was successfully used to repair cartilage defects in rats. The dual-crosslinked hydrogel demonstrated superior mechanical properties, exhibiting a compressive strength of approximately 245 kPa and a compressive strain of 60% at 1% sericin content, with water content ranging from 85 to 88% and porosity exceeding 20%. These characteristics provided adequate space and nutrient transport conditions for cellular growth. In in vivo cartilage defect repair experiments, the dual-crosslinked hydrogel group formed regenerated tissue with molecular architecture analogous to native cartilage after 4 weeks of implantation. The International Cartilage Repair Society score of this group was significantly higher than those of the control group and the sodium alginate hydrogel-only group, confirming its exceptional cartilage regeneration capability.^[46]^ To meet the mechanical strength requirements for bone tissue repair and enhance the material’s deformation resistance and structural support capacity, a study developed a hydrogel by integrating sericin with graphene oxide (GO) and photo-crosslinkable methacryloyl groups. This hydrogel exhibited sufficient mechanical robustness, with compressive moduli of 17, 34, and 43 kPa for SMH, SMH/GO-1, and SMH/GO-2, respectively. Furthermore, it effectively repaired bone defects by inducing directional migration and osteogenic differentiation of autologous BMSCs. In the SMH/GO-2 group, both protein expression (Osteocalcin/Ocn, Collagen Type I/Col I, and Runt-related transcription factor 2/Runx2) and mRNA levels of BMSCs were significantly elevated compared to the control group. The system synergistically integrated the biomechanical enhancement of the material with the inherent bioactivity of sericin.^[47]^ These composite hydrogels, which can mimic the microenvironment and porosity of a specific extracellular matrix, can be modified with other chemicals to give them different mechanical strengths and better adapt to tissue engineering needs.

### 3.3. Recombinant protein materials

In recent years, genetically modified cell-free biomaterials have shown excellent performance in tissue engineering and wound healing.^[48]^ Sericin, a biologically produced protein, is more promising for modifying protein materials through genetic engineering. Modified proteins would bind and fuse different functional domains, merging cell adhesion and migration, mechanical properties and antimicrobial factors to achieve multifunctional biomaterial systems, eliminating the side effects of physical or chemical binding of materials.^[49]^ In recent studies, researchers have been able to control the expression region of recombinant protein genes in sericin and have been able to control the localization of synthetic proteins in sericin^[50]^ to produce substances that are more readily available to us. Matrix metalloproteinase degradable sequences have been inserted into different regions of the polymer backbone to make more robust hydrogels with more excellent intermolecular interactions.^[51]^

In addition to structural modifications, genetic engineering of sericin with functional exogenous proteins can enhance their biological functions and further expand their applications in tissue engineering, e.g., human growth factors were doped into sericin to prepare hydrogels, which continuously released growth factors to promote cell proliferation and wound healing.^[52]^ Sericin produced by genetically modified *Bifidobacterium bifidum* with growth factors, which promoted wound healing ability, was significantly enhanced.^[53]^ Hydrogels prepared using transgenic silkworms producing sericin-functionalized with human platelet-derived growth factor (PDGF-BB) held osteoblastic differentiation of MSCs in vitro and in vivo to promote bone tissue regeneration.^[54]^ Thanks to the advantages of transgenics, we can modify and transform the protein substances we need, making our research in materials more diverse and more precisely targeted. However, genetic engineering is a double-edged sword; breaking the laws of natural inheritance may lead to the contamination of normal genes, and the appropriate use of this technology will allow it to continue to benefit humanity.

### 3.4. Sericin-based materials in bone regeneration

#### 3.4.1. Sericin-based hydrogels

In bone regeneration engineering, scaffold materials need sufficient support and stiffness with appropriate porosity to meet the requirements of bone defect regeneration. Sericin can be used as a scaffold material in bone tissue engineering. Still, as a globular protein, the mechanical strength of scaffolds synthesized from it is insufficient, so we need to strengthen the performance of scaffolds to support bone regeneration using modification. Firstly, adding different chemical components to make the material harder is an effective method.

As a bone tissue engineering material, hydrogel has the characteristics of high plasticity, is close to cancellous bone, and mimics extracellular matrix, etc, sericin, which has excellent bioactivity, is an ideal raw material for hydrogel, and the modification of sericin-based hydrogel can significantly improve the mechanical strength of the material. A study developed a novel sericin methacrylate (SMH)/GO composite hydrogel by incorporating methacrylic acid into a sericin solution combined with GO, followed by photoinitiator crosslinking. The GO component endowed the hydrogel with tunable mechanical strength, achieving a compressive modulus of 43 kPa, which is comparable to that of natural bone tissue. This hydrogel exhibited excellent biocompatibility, biodegradability, cell adhesion, and pro-proliferative effects. Furthermore, it enhanced osteogenesis by modulating the migration and osteogenic differentiation of autologous BMSCs, significantly upregulating osteogenic marker expression and improving neobone formation efficiency (bone regeneration rate > 90% at 12 weeks).^[47]^ Similarly an injectable alginate/sericin/graphene (Alg/Ser/GO) hydrogel based on an alginate-tyramine hydrogel framework and HRP/H with good mechanical properties and tightly controlled degradation behavior upon release of the sericin and GO provided an environment of reduced host immune response, decreased inflammation, M2 macrophage infiltration, and greater polarization, which activated and induced the differentiation of BMSCs into osteoblasts. GO in the prepared hydrogels further promoted osteogenic differentiation and bone regeneration.^[55]^ A newly developed porous hydrogel, fabricated by lyophilizing a blend of sericin, gelatin, and carrageenan, provides a microenvironment conducive to osteoblast attachment and proliferation. By matching the mechanical strength of natural bone and activating signaling pathways to recruit autologous BMSCs and induce their differentiation, this hydrogel achieves highly efficient bone regeneration (regeneration rate > 90% at 12 weeks).^[56]^ Incorporating sericin into a collagen-based hydrogel system and inducing hydroxyapatite (HAp) deposition on its surface, comparative analysis revealed that the addition of sericin significantly enhanced the metabolic activity of MSCs in the sericin 40% hydrogel compared to pure I-DC hydrogel under basal culture conditions (Days 7, 14, and 21; *P* < .05). The highest metabolic activity in osteogenic medium was observed on Day 14. Alizarin Red staining demonstrated markedly higher mineralization levels in the sericin 40% hydrogel than in the pure I-DC hydrogel under osteogenic conditions. Scanning electron microscopy further revealed that the sericin 40% hydrogel exhibited larger and more densely distributed mineralized particles, indicating substantially enhanced osteogenic capacity of the composite hydrogel.^[57]^

The regulation of growth factors and drugs plays a crucial role in tissue engineering, and the delivery of growth factors and drugs is one of the essential functions of tissue engineering scaffolds: sericin-based hydrogels can be used as the basis for drug delivery systems in bone tissue engineering. Biomaterials can be used for loading and sustained release of growth factors and drugs. An interpenetrating polymer network hydrogel with sericin and alginate, where the proportion of alginate can be adjusted to adjust the strength, and a high content of sericin can show stability during degradation, can be widely used as a platform for drug delivery in tissue engineering.^[58]^ Sericin/polyvinyl alcohol (PVA) hydrogel loaded with ciprofloxacin has a solid ability to promote bone tissue regeneration sericin and PVA ratios up to 75:25 and 50:50, which has a wide range of promising applications in the treatment of osteomyelitis and bone regeneration.^[59]^ Drug delivery relies on the stability and slow degradation of biomaterials. The long-term release of biofactors is more favorable to the reconstruction of bone tissues. The modified sericin-based hydrogels can be stably degraded in vivo and release the growth factors and drugs they carry.

#### 3.4.2. Sericin-based biofilms

Guided tissue regeneration membranes are most commonly used to inhibit fibrous tissue’s inward growth and create a suitable space for alveolar bone regeneration.^[60]^ Sericin is also often used as a biofilm with good biological properties in guiding bone regeneration.^[61,62]^ In another study, a sericin-hydroxyapatite (Ser-HAp) composite nanomaterial was fabricated via a biomineralization approach. The synthesis protocol involved preparing a 4% PVA solution by dissolving 4 g of PVA in 100 mL of deionized water under continuous stirring at 100°C for 1 hour. A 1% sericin solution was then blended with the 4% PVA solution at a 1:1 volume ratio, followed by homogenization at 80°C for 30 minutes. Ser100-HAP particles were subsequently incorporated into the mixture at concentrations of 0, 10, 50, and 100 μg/mL, with additional stirring for 30 minutes. The resulting composite was transferred into 24-well plates and subjected to 3 freeze-thaw cycles (−20°C freezing followed by room-temperature thawing). The samples were lyophilized for 24 hours to form membranes. Osteogenic induction assays demonstrated that sericin enhanced the osteogenic differentiation of human periodontal ligament stem cells by upregulating the expression of osteoblast-related markers, including alkaline phosphatase (ALP), *Runx2*, OCN, and osteopontin.^[63]^ Sericin was chemically bonded to cellulose acetate membranes via APTS and glutaraldehyde to form a novel functionalized biofilm, and membrane degradation studies in physiological pH solutions demonstrated that the CA-APTS-GA-SER membrane mass was reduced by only 26% within 3 months and that the CA-APTS-GA-SER membranes elicited a good osteoclastogenic response in terms of cell adhesion, viability, and proliferation.^[64]^ HAp deposition onto sericin films significantly increased its biomineralization capacity and ability to promote cell adhesion and proliferation.^[65]^ The guided tissue regeneration membrane made using sericin had excellent mechanical properties and stability and showed excellent osteogenic differentiation, proliferation ability, and new bone regeneration. Fibroblast growth factor was doped into a sericin membrane and implanted into rat cranial defects. The membrane exhibited stable drug release and could accelerate bone tissue remodeling and wound healing.^[66]^ For biofilm applications, sericin has excellent biological properties and shows great potential.

## 4. Biological effects of sericin osteogenesis

### 4.1. Promoting osteoblast proliferation

Osteoblasts are important seed cells responsible for osteogenesis and mineralization in bone tissue engineering. This cell type is derived from MSCs and can secrete collagen fibers, extracellular matrix, and related enzymes to promote osteogenesis and mineralization.^[67]^ It has been found that filaggrin promotes bone tissue’s growth and repair mainly by promoting osteoblasts’ proliferation and differentiation. Sericin extracted from non-mulberry silkworm cocoons has been demonstrated to enhance the osteogenic differentiation of BMSCs. The sericin/hydroxyapatite (AS/HAp) composites (AS_1_ and AS_5_) significantly promoted the proliferation of human BMSCs, with AS_5_ exhibiting a higher proliferation rate than AS_1_ and control groups (tissue culture plastic/TCP and pure AS) at 5 days. Both AS_1_ and AS_5_ showed significantly elevated ALP activity compared to non-mineralized AS, while AS_5_ demonstrated superior osteoinductive capacity.^[68]^ Furthermore, sericin extracted from *Bombyx mori* cocoons has been demonstrated to enhance osteogenic differentiation of bone marrow stem cells (BMSCs). The experimental procedure involved cutting silkworm cocoons into fragments, which were then boiled for 10 minutes to solubilize the sericin protein (BS). After centrifugation, the supernatant was collected and subsequently dialyzed using dialysis membranes. The BS solution was immersed in 1.5-fold simulated body fluid (1.5 × SBF, pH 7.25) and mineralized at 37.2°C for 5 days. The resulting BS/HAp composite (BS_5_) exhibited significant promotion of BMSCs proliferation (*P* < .01). ALP activity assays revealed that the BS_5_ group demonstrated the highest ALP activity among all experimental groups (*P* < .01).^[69]^ A study reported an unmodified sericin that could encourage the proliferation of osteoblasts on the one hand and effectively inhibit the formation of bacterial biofilm on the other hand, which could be used to treat osteomyelitis.^[70]^ Some researchers produced a membrane scaffold cast through arginine self-assembled sericin nanofibers, which could induce osteoblast differentiation of BMSC.^[71]^ When sericin was used to modify titanium surfaces, it was found that sericin could increase bone signals such as bone sialoprotein, OCN, and ALP.^[72]^ These applications of sericin in bone tissue materials effectively promoted the proliferation of osteoblasts.

**Figure 1.**
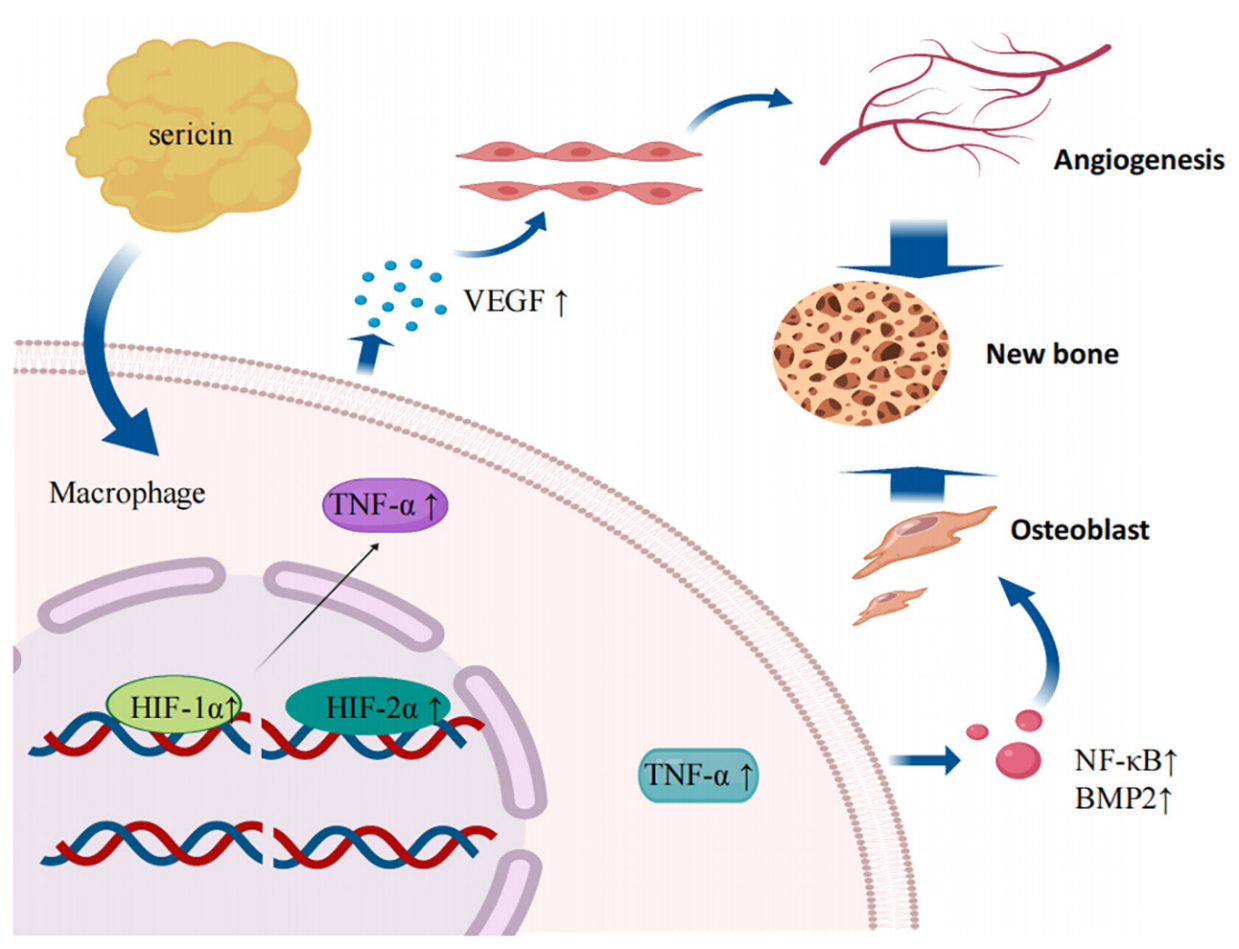
The physiological function of sericin within the body’s framework. BMP-2 = bone morphogenetic protein-2, TNF-α = tumour necrosis factor-α, VEGF = vascular endothelial growth factor.

Regarding how sericin affects the function of osteoblasts in achieving bone regeneration, it has been explored that the role of sericin may be related to the release of bone morphogenetic protein-2 (BMP-2) from macrophages. Li Zhihai et al, with collagen made into a scaffold piggybacking sericin into sericin-based bone scaffold material, constructed a rat bone defect model; the results found that sericin can activate macrophages to release BMP-2, recruit osteoblasts and other bone-forming cells, and promote the healing of bone tissue.^[73]^ Gels incorporating sericin promote the expression of ALP and Runx2 in MSCs. Isolated sericin increased the expression of BMP-2 mRNA in mouse macrophages (RAW264.7) via a TLR-mediated pathway, which in turn enhanced the osteogenic activity of the cells.^[74]^ Moreover, this enhancement appeared to be associated with the β-folding of sericin, and 4-hexylresorcinol-pretreated sericin enhanced the β-sheet structure of sericin, with significantly higher expression of BMP-5 and runx2.^[75]^ It has been found that the matrix component of sericin-promotingbone formation can be explained by the tumour necrosis factor-α (TNF-α) mediated pathway and that high concentrations of TNF-α increase gene expression of BMP-2.^[76]^ It has been shown that sericin promotes M2 polarization and migration via the NF-κB and MAPK pathways and that M2 polarization of macrophages induces osteogenic differentiation of BMSC via several secreted cytokines.^[55]^ In conclusion, sericin may increase intracellular TNF-α expression and, consequently, BMP-2 expression; on the other hand, sericin induces osteogenic differentiation by stimulating macrophage polarization (e.g. Fig. 1).

### 4.2. Promote angiogenesis

In addition to the need for seed cells such as osteoblasts, the growth of new blood vessels is also critical for repairing bone defects. Generally, blood vessels grow into the defect before bone tissue, providing oxygen and transporting nutrients and metabolic wastes for bone repair. Song Yan et al prepared sericin hydrogel in a mouse myocardial ischemia model that can improve local blood vessel formation, increase blood vessel density and increase blood vessel diameter; sericin can promote the proliferation and migration of endothelial cells.^[77]^ GO nanomaterials functionalized with sericin, added to seed eggs and incubated for 3 hours showed significant vascular proliferation in chicken embryos compared to controls.^[78]^ A study of sericin and collagen-treated graphene materials found that there was also a substantial proliferation of blood vessels in sericin-collagen-treated chicken embryos.^[79]^ Hybrid hydrogels made of sericin proteins were implanted into rats in subcutaneous in vivo experiments, and the angiogenesis rate in the tissues was higher than in the control group after incubation.^[80]^ The cream made of sericin and acacia resin was applied daily for 10 days on a rat wound model, and histological examination showed a large amount of angiogenesis.^[81]^ A composite made of sericin and carboxymethyl chitosan significantly promoted angiogenesis in a mouse wound model.^[82]^

Angiogenesis is mainly regulated by vascular endothelial growth factor (VEGF). Experiments have shown that sericin treatment presented as cells improved the mRNA expression of VEGFa through the Ras-MEK-ERK signaling pathway, thereby promoting neovascularization.^[83]^ Youjin Zhao et al inversely verified that sericin-induced angiogenesis increased VEGF expression in RAW264.7 cells through a hypoxia-inducible factor-mediated pathway by cytokine inhibitors.^[84]^ Then, sericin can increase the expression of VEGF and increase angiogenesis in tissues (Fig. 1).

### 4.3. Promoting biomineralization

The structural composition of sericin itself promotes biomineralization and has shown great potential in tissue engineering. Early studies demonstrated that HAp mineralization on sericin surfaces, followed by assembly into spherical composites, resulted in the formation of spherical HAp crystals on the sericin (AS) film. With increasing immersion cycles, the crystal density and aggregation degree were significantly enhanced. Cross-sectional analysis revealed a continuous mineralized layer tightly integrated with the AS film. Fourier-transform infrared spectroscopy of the mineralized AS film exhibited characteristic peaks at 560 and 602 cm^−1^ (ν_4_ vibrations of PO_4_^3−^) and 1030 cm^−1^ (ν_3_ vibrations of PO_4_^3−^), confirming the successful formation of apatite.^[65]^ A subsequent study found that sericin and HAp nucleated through electrostatic interactions and hydrogen bonding.^[85]^ In a new study, inorganic nanomaterials were deposited on the surface of sericin by taking advantage of the unique molecular structure of sericin, which is rich in acidic amino acids that result in multiple negatively charged sites, and this strategy resulted in a biomimetic mineralized material with highly efficient osteogenic capacity and other biological effects.^[86]^ Sericin extracted through hot water can form β-sheet side-chain groups, and this unique structure attracts ions and thus promotes the mineralization of nano-ions, based on which Ser-HAp nanoribbons were formed with excellent osteogenic properties.^[87]^ Similarly, based on the properties of sericin to interact with calcium ions, a study enhanced the mineralization of calcium phosphate by sericin, resulting in a material with excellent cellular activity, which is ideal for application in complex tissue regeneration.^[88]^ The biomineralization properties of sericin can form a stable structure for nanoparticles with excellent osteogenic activity.

Biomineralized materials derived from sericin have been shown to enhance cellular proliferation and differentiation, thereby promoting osteogenesis. A study demonstrated that sericin, when combined with HAp, mediates the nucleation of HAp into nanoneedle-like structures. At a sericin concentration of 0.5 mg/mL, HAp formed nanoneedles (20–40 nm in length and 3–5 nm in width), whereas at 8 mg/mL, HAp exhibited significant aggregation. The formation of such biomineralized protein-based biomaterials suggests that sericin mineralization can stimulate cellular adhesion and proliferation.^[89]^ A GO-based nano sericin/HAp composite hydrogel stimulates bone formation through the synergistic effect of sericin immunomodulation of macrophage polarization and direct osteogenesis induction by nHAP. The ALP activity in the sericin-treated group (1 mg/mL, 7 days) was significantly elevated to 25.3 ± 2.1 U/mg protein, representing a 109% increase compared to the control group (12.1 ± 1.5 U/mg protein).^[90]^ The HAp/sericin biofilm exhibited mechanical strength and cell viability.^[91]^ Another study fabricated Ser-HAp composite nanomaterials using a biomimetic mineralization approach. The preparation protocol involved dissolving 4 g of PVA in 100 mL deionized water to prepare a 4% PVA solution, which was stirred at 100°C for 1 hour. A 1% sericin solution was then mixed with the 4% PVA solution in a 1:1 ratio and stirred at 80°C for 30 minutes. Ser100-HAp particles (0, 10, 50, or 100 μg/mL) were added to the mixture, followed by additional stirring for 30 minutes. The resulting blend was poured into 24-well plates, subjected to 3 freeze-thaw cycles (−20°C freezing and room-temperature thawing), and lyophilized for 24 hours to form membranes. Osteogenic induction assays demonstrated that sericin promoted the osteogenic differentiation of human periodontal ligament stem cells by activating the expression of osteoblast-related genes, including ALP, Runx2, OCN, and osteopontin.^[63]^

## 5. Discussion

This review provides past and current insights into sericin in bone tissue engineering. Sericin, an industrial waste from silk production, is a natural substance with potential for a wide range of applications in bioengineering. Sericin is biocompatible, nontoxic and degradable, and its compatibility with cells is also demonstrated by its ability to promote cell proliferation, which is crucial for its potential application in regenerative engineering. The chemical structure of sericin makes the sericin effect modify the surface of the material, which can significantly enhance the activity of biomaterials, and the synergistic effect of sericin improves the function of biomaterials to be enhanced or expands. In addition, the protein chemical structure of sericin is also favorable for sericin to be used as the primary material of biomaterials, which can be modified by various substances to make valuable sericin in multiple fields of tissue engineering.

In ongoing research, sericin may play a role in bone tissue engineering, bone repair and bone regeneration. On the one hand, sericin can promote the proliferation and differentiation of osteoblasts, such as promoting the polarization of macrophages to secrete various types of growth factors, thus promoting osteogenesis; on the other hand, sericin can increase the expression of vascular endothelial factor through cellular pathways to promote angiogenesis, which is very important for bone tissue regeneration.

Sericin has a protein chemical structure, and after modification with various substances, it has properties such as plasticity, cancellous bone-like nature, extracellular matrix simulation, etc, which makes it ideal as a raw material for bone tissue engineering, and it can be made into hydrogel, biofilm, etc, and can be piggybacked on drugs. The study found that the biomineralization properties of sericin can form a stable structure for the nanoparticles and have excellent osteogenic activity. Of course, the current study is limited to investigating the osteogenic effect of sericin, and future research will further clarify its specific role and mechanism in osteogenic applications.

## 6. Conclusion

This article reviews some of the past and current applications of sericin in bone tissue engineering. Through chemical modification, sericin can be made into hydrogels, biomaterials, and other materials for bone tissue engineering applications. It also describes how sericin can promote the biological effects of bone regeneration. Overall, sericin has potential applications in bone tissue engineering.

## Author contributions

**Conceptualization:** Rui Wang, Zhihui Liu.

**Data curation:** Jiatong Zou.

**Formal analysis:** Rui Wang, Zhihui Liu.

**Investigation:** Rui Wang.

**Methodology:** Rui Wang.

**Project administration:** Zhihui Liu.

**Resources:** Rui Wang.

**Supervision:** Jiajun Xin, Yifei Dai, Xiantao Chen, Yushen Li, Bowei Wang.

**Validation:** Rui Wang.

**Writing – original draft:** Rui Wang.

**Writing – review & editing:** Jiatong Zou, Jiajun Xin.
